# Supplementary material for: The impact of COVID-19 on the physical activity and sedentary behaviour levels of pregnant women with gestational diabetes
Source: PLoS One. 2021 Aug 20;16(8):e0254364. doi: 10.1371/journal.pone.0254364 (PMC8378749; doi:10.1371/journal.pone.0254364)
Supplement: Attachment — Submitted filename: Response to Reviewers.docx [file pone.0254364.s001.docx]

RE: “The impact of COVID-19 on the physical activity and sedentary behaviour levels of pregnant women with gestational diabetes.” Response to Reviewers 27^th^ April 2021.

| **Reviewer’s comments** | **Response** |
| --- | --- |
| Please edit and focus the introduction which is rather long at present | Introduction has been edited and reduced in length |
| Educational qualifications - suggest amend to indicate highest qualification gained as the results are not interpretable at present. | Changed to degree/no degree |
| How would you advise researchers to address the issue of responder bias and the difficulty in getting an ethnically representative sample for this sort of study in future? | Suggestions added Section 6: strengths and limitations |
| Table 1: the column headings are misleading – I would suggest maternal demographics rather than age for the first column. Why are both range of age and mean values included? Are the data normally distributed? | Table simplified. Maternal characteristics heading added to first column. Range removed. |
| It may be better to consider calculating the p value on two simple categorical alone eg qualifications vs no qualifications, and/or higher degree versus no higher degree as the number of variables included in the current analysis renders a high probability that the statistical significance could be related to chance. The same comment applies to table 2 and treatment for GDM. | Table simplified. Changed to degree/no degree. P value given between the two categories.  Table 2 GDM management changed to diet/medication |
| Tables 3 and 5: these tables are confusing and the headings need explanation | Tables simplified and headings for tables 3 and 5 explained |
| Lines 276-280: The mean gestational age of women in the sample is 22.1 weeks. The mean GA that women were diagnosed with GDM is 24.1 weeks. Do the authors have a comment on this discrepancy? | In line with the NICE guidelines (NG3) women who have previously had GDM should be offered early self monitoring of blood glucose or a 75g 2h Oral Glucose Tolerance Test as soon after booking as possible. Therefore these results suggest a larger number of women in the sample had GDM in a previous pregnancy and were therefore diagnosed earlier than the 24-28 week standard OGTT. This is in keeping with the fact that 62% of the sample were multiparous. |
| Discussion: caution needs to be exercised in terms of over-stating the results. While the results indicate that women’s levels of activities dropped during covid, these are questionnaires asking women about past behaviours i.e. recall bias needs to be considered. Furthermore, at what point were women taking “pre-covid times”? Would this potentially not have included much earlier gestational age when physical activity would have been much less limited? | It has been highlighted in section 6 Strengths and limitations that self-reporting and reliance on recall can result in inaccuracies. In addition, it has been highlighted that as women were self-reporting activity levels at both time points reporting inaccuracies are likely to be non-differential and therefore not affecting the general level of decline seen.  In addition, the PA levels by trimester have been added to section 3.2 physical activity levels, showing that PA levels were lower in T1 and increased in T2. |
| Lines 77-79 are not clear: do the authors mean “reported cases of severe covid”? | Removed |
| Line 113: Statement is narrow. Women of other non-white European ethnic groups such as Arab/ South East Asian/ Hispanic/ Black African Caribbean are also at risk of developing gestational diabetes. | Line 149-150 made broader |
| Lines 139-141: evidence already explained earlier in the text therefore does not need to be repeated. | Earlier evidence/explanation removed to avoid repetition |
| Power calculation for sample size: what outcome was the power calculation based on and what statistical analysis was used to reach the sample size? | The outcome the power calculation was based on was the number of women diagnosed with GDM. There were 731,213 births in the UK in 2018. It is estimated that approximately 4.4% of women in the UK have GDM. This would mean approximately 32,173 women are diagnosed with GDM annually in the UK. Based on a power calculation with a population size of 32,173, at a 95% confidence level and 5% error, it was calculated that 380 women would need to complete the survey to provide representative results. |
| Lines 297- 308: are all the questions that women were asked included in this section? | No this is not all the questions which were asked but the questions relevant to this analysis. |
| Line 292: This statement is confusing. Do the authors mean that x proportion of women adhered to PA guidelines according to the results of the questionnaire? Or is the statement inferred from the mean number of days that women exercised for over 30 minutes? If the latter, how they arrived at the calculation needs to be explained. | Further explanation to how the percentage was arrived at is given. |
| Line 376/377. This is not necessary to include. If women say they are more sedentary it would follow that they are likely to be less physically active | Sedentary time and PA are two different behaviours. An individual can have increased their sedentary time but also have increased with PA, therefore looking at both of these behaviours separately is important. |
| Lines 340-343. Proportions here are of more value than reporting “x | Proportions are given in the original manuscript |
| Physical activity tends to reduce for pregnant women as pregnancy progresses due to the physiological changes of pregnancy. How this observation affected their results was not clearly addressed in either the introduction or the discussion sections of the manuscript. | Information on PA levels during each trimester has been included in section 3.2 |
| The introduction is a bit too expansive and can be modified to become more concise based on the stated aims and objectives of the research. | Introduction has been edited and made more concise. |
| In your Methods section, please include additional information about your dataset and ensure that you have included a statement specifying whether the collection method complied with the terms and conditions for the website. | A statement that data was collected within the terms and conditions of the websites has been added to the methods section. Along with details that the full questionnaire and data set are available online. |
